# Structural and functional characterization of a conserved cryptic epitope on SARS-CoV-2 spike S2 subunit

**DOI:** 10.1371/journal.ppat.1014391

**Published:** 2026-08-03

**Authors:** Ling Zhou, Ching-Lin Hsieh, Sarah R. Leist, Emily Happy Miller, Andrew P. Horton, Sophie R. Shoemaker, Elizabeth C. Gardner, John M. Powers, Daniel R. Boutz, Michael L. Mallory, Connor M. Mullins, Nicole V. Johnson, Alexandra L. Tse, Albert Wang, Kamyab Javanmardi, Lily E. Adams, Jonathan Schisler, Susan Marqusee, Kartik Chandran, Ralph S. Baric, Jimmy D. Gollihar, Jason S. McLellan

**Affiliations:** 1 Department of Molecular Biosciences, The University of Texas at Austin, Austin, Texas, United States of America; 2 Department of Epidemiology, University of North Carolina at Chapel Hill, Chapel Hill, North Carolina, United States of America; 3 Department of Microbiology and Immunology, Albert Einstein College of Medicine, Bronx, New York, United States of America; 4 Department of Medicine-Infectious Diseases, Albert Einstein College of Medicine, Bronx, New York, United States of America; 5 Department of Pathology & Genomic Medicine, Antibody Discovery and Accelerated Protein Therapeutics, Houston Methodist Research Institute, Houston, Texas, United States of America; 6 Department of Molecular and Cell Biology, University of California, Berkeley, Berkeley, California, United States of America; 7 McAllister Heart Institute, University of North Carolina at Chapel Hill, Chapel Hill, North Carolina, United States of America; 8 Department of Pathology & Lab Medicine, University of North Carolina at Chapel Hill, Chapel Hill, North Carolina, United States of America; 9 Department of Pharmacology, University of North Carolina at Chapel Hill, Chapel Hill, North Carolina, United States of America; 10 Department of Chemistry, University of California, Berkeley, California, United States of America; 11 Chan Zuckerberg Biohub, San Francisco, California, United States of America; 12 California Institute for Quantitative Biosciences, University of California, Berkeley, Berkeley, California, United States of America; The University of Texas Medical Branch at Galveston, UNITED STATES OF AMERICA

## Abstract

Severe acute respiratory syndrome coronavirus 2 (SARS-CoV-2) has undergone extensive evolution since its emergence in 2019, underscoring the continuous need for vaccines and therapeutics effective against multiple variants of concern (VOCs). The S2 subunit of the viral spike (S) glycoprotein is highly conserved among sarbecoviruses, making it an attractive target for broadly protective countermeasures. To elucidate the S2 antigenic landscape, we employed yeast surface display to isolate S2-targeted antibodies from COVID-19 convalescent donors. Biophysical characterization revealed that these S2 apex-directed antibodies preferentially bind to open spike conformations and a stabilized S2 construct but not to the closed, trimeric prefusion spike. Cryo-electron microscopy structures defined a cryptic epitope encompassing the upper helix and fusion peptide proximal region on S2. This epitope is conserved among sarbecoviruses but remains largely occluded in the closed prefusion conformation of the spikes. As a result, the antibodies exhibited weak neutralization activity against SARS-CoV-2 pseudoviruses, failed to neutralize authentic viruses, and did not provide protection in a lethal mouse challenge model using a mouse-adapted SARS-CoV-2 strain. These findings highlight a non-neutralizing epitope on S2 capable of eliciting antibodies during SARS-CoV-2 infection in humans and provide valuable reagents for probing S2 conformational dynamics and optimizing S2-based vaccine antigens.

## Author summary

The rapidly evolving severe acute respiratory syndrome coronavirus 2 (SARS-CoV-2) continues to challenge vaccines and antibody therapeutics that primarily target the variable S1 subunit of the spike (S) glycoprotein. In contrast, the fusion machinery, the S2 subunit, is more conserved among sarbecoviruses and represents a potential target for broadly reactive antibodies. Here, we used yeast surface display to isolate S2-directed antibodies from COVID-19 convalescent donors and characterized their structural and functional properties. Biophysical analyses showed preferential binding to open spike conformations and a stabilized S2 construct, but not to the closed prefusion trimer. Cryo-electron microscopy structures revealed a conserved, cryptic epitope spanning the upper helix and fusion peptide–proximal region (FPPR) that is largely occluded in the closed spike. Despite cross-reactivity, these antibodies weakly neutralized SARS-CoV-2 pseudoviruses, failed to neutralize authentic virus, and did not protect mice in a lethal challenge model. Collectively, our findings define a conserved but non-neutralizing cryptic S2 epitope targeted by infection-elicited antibodies and provide reagents to probe spike conformational dynamics and inform the rational design of S2-based immunogens that minimize exposure of non-protective epitopes.

## Introduction

Severe acute respiratory syndrome coronavirus 2 (SARS-CoV-2), a betacoronavirus first identified in Wuhan, China, in 2019, is the causative agent of coronavirus disease 2019 (COVID-19). As of October 2025, the COVID-19 pandemic has resulted in over seven million deaths globally [1]. The urgent need to mitigate this crisis spurred the rapid development of vaccines, culminating in the emergency use authorization of four COVID-19 vaccines in the United States [2]. However, the emergence of variants of concern (VOCs) has triggered recurrent surges and diminished vaccine effectiveness [3–6], necessitating reformulated vaccines to broaden protection. Despite these efforts, there remains an ongoing need for broadly effective therapeutics to combat this evolving virus.

The SARS-CoV-2 spike (S) protein, the sole viral antigen incorporated into the four authorized COVID-19 vaccines, is a class I viral fusion protein comprising two subunits, S1 and S2 [7]. The S1 subunit contains the receptor-binding domain (RBD), which engages the host receptor angiotensin-converting enzyme 2 (ACE2) [8], as well as the N-terminal domain (NTD) and two smaller subdomains. The S2 subunit facilitates membrane fusion and comprises the fusion peptide, transmembrane domain, and two heptad-repeat domains (HR1 and HR2). Structurally, three S1 subunits form a fusion-suppressive cap over a trimeric S2 subunit, which is maintained in a spring-loaded, metastable prefusion conformation. Upon ACE2 engagement, conformational rearrangements in the spike protein are triggered [9,10], leading to S1 shedding and S2 refolding into a highly stable post-fusion conformation.

Potently neutralizing antibodies predominantly target epitopes on the RBD and NTD of the S1 subunit, and several have been deployed as COVID-19 therapeutics [11–15]. However, the frequent emergence of escape mutations within S1 in VOCs has significantly reduced the efficacy of many S1-targeted antibodies [16–18]. In contrast, the S2 subunit is highly conserved among VOCs and across other SARS-related coronaviruses (sarbecoviruses), making it a compelling target for antibodies with broad resistance to viral escape. Broadly neutralizing antibodies have been identified that recognize two key antigenic sites on the S2 subunit: the fusion peptide [19–22] and the stem helix preceding heptad repeat 2 (HR2) [23–25]. Additionally, non-neutralizing antibodies have been identified that bind the apex of the S2 subunit [26–29]. Despite these advances, much of the S2 antigenic landscape remains uncharacterized, and additional epitope targets on S2 may yet be defined.

To further elucidate the human antibody response to the S2 subunit, we isolated two cross-reactive, SARS-CoV-2 S2-specific antibodies from COVID-19 convalescent donors using yeast surface display (YSD). Cryo-electron microscopy (cryo-EM) structures of these antibodies in complex with a stabilized S2-only antigen [30] elucidated the molecular basis of recognition and revealed a cryptic epitope. Functional analyses, including pseudovirus and authentic virus neutralization assays, along with *in vivo* protection studies in mice, were conducted to assess therapeutic potential. The results of these studies advance our understanding of humoral responses targeting the conserved S2 subunit, provide reagents to probe spike protein conformational dynamics, and inform the rational design of next-generation COVID-19 vaccines.

## Results

### S2 prefusion trimer apex-directed antibody isolation from COVID-19 convalescent donors using yeast surface display

We previously reported the isolation of potent RBD- and NTD-specific neutralizing antibodies targeting the SARS-CoV-2 (Wuhan-1) spike protein from three individuals infected during the first wave of the COVID-19 pandemic and sampled 11–12 days post-symptom onset [31–33]. These antibodies were identified via three rounds of yeast surface display (YSD) using the prefusion-stabilized SARS-CoV-2 spike ectodomain (S-2P) as the selection probe [31,32,34]. During characterization of the IgG repertoire from these donors, we identified a subset of antibodies that did not bind to soluble RBD or NTD proteins [31]. Notably, the VH3–30 heavy chain gene was overrepresented, particularly among antibodies with undefined specificities (S1 Fig). Enzyme-linked immunosorbent assay (ELISA) binding assays using a prefusion-stabilized S2-only antigen [30,35] (‘HexaPro SS’) revealed that many of these VH3–30 antibodies specifically recognized the S2 subunit (S2 Fig).

**Fig 1:**
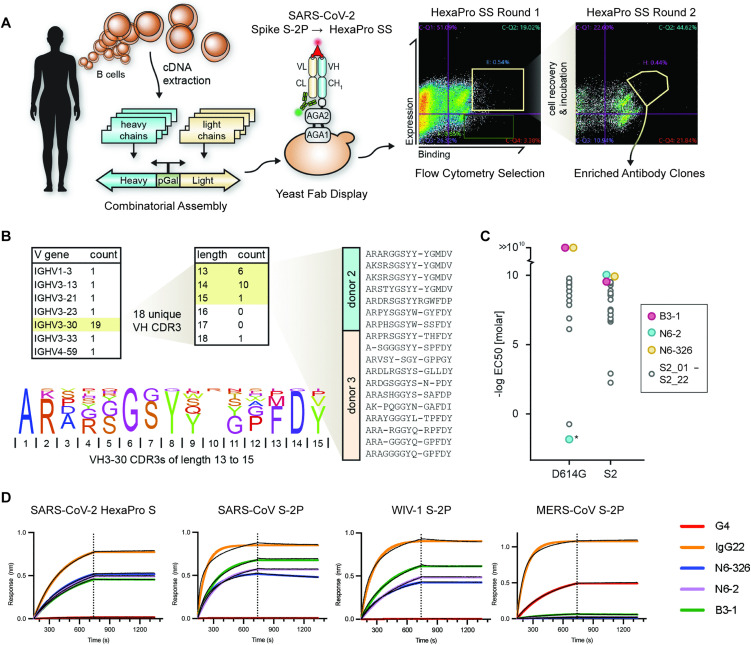
Isolation and characterization of S2 apex-directed antibodies from convalescent donors using yeast surface display. (**A**) Schematic overview of the antibody isolation strategy from convalescent plasma, employing yeast surface display of Fab libraries and flow cytometry-based cell sorting. **(B)** V gene usage among 25 unique VH-VL pairs identified through sequencing, highlighting VH3-30 enrichment and the diversity of H-CDR3 lengths and sequences. **(C)** ELISA binding of all 25 antibodies to SARS-CoV-2 HexaPro D614G spike ectodomain and the stabilized S2-only construct, HexaPro SS. An asterisk indicates poor curve fitting (see S2 Fig for raw binding data). **(D)** BLI binding analysis of selected S2 apex-directed antibodies (N6-2, N6-326, and B3-1) to spike ectodomains from diverse betacoronaviruses. G4 [36] (MERS-CoV S-specific control antibody) and IgG22 (positive control antibody targeting the helical stalk of the spike protein) were included. Experimental curves are shown in black; fitted curves are overlaid in color.

Given the strong S2 reactivity observed for VH3–30–encoded antibodies, we employed YSD with HexaPro SS as a selection probe to isolate additional S2-specific antibodies from the previous round 3 outputs. Following two additional rounds of HexaPro SS selection (Fig 1A), sequencing of paired VH-VL genes revealed 25 unique VDJ rearrangements, of which 19 utilized the VH3–30 germline gene. These VH3–30 antibodies encoded 18 distinct H-CDR3 sequences, ranging from 13 to 15 amino acids in length (Figs 1B; S1). Somatic hypermutation rates ranged from 0.3% to 6.1% (median 1.0%) for the heavy chain V-gene, indicating that the VH3–30 mAbs likely originated during the primary response to SARS-CoV-2. ELISA assays showed robust binding of these antibodies to both the SARS-CoV-2 spike ectodomain D614G containing HexaPro substitutions [35] and to HexaPro SS [30] (Fig 1C**).** Based on their strong binding profiles, three antibodies—N6-2, N6-326, and B3-1—were selected for further analysis. Biolayer interferometry (BLI) data demonstrated that these antibodies bound comparably to spike ectodomains from multiple sarbecoviruses, including SARS-CoV-2, SARS-CoV, and WIV-1, but failed to bind the MERS-CoV spike ectodomain (Fig 1D). These results suggest that the epitopes recognized by these S2-specific antibodies are conserved across sarbecoviruses but are not preserved in non-sarbecovirus betacoronaviruses.

### S2 prefusion trimer apex-directed antibodies bind to open-interface spikes or isolated S2 subunits but not to closed spikes

**Fig 2:**
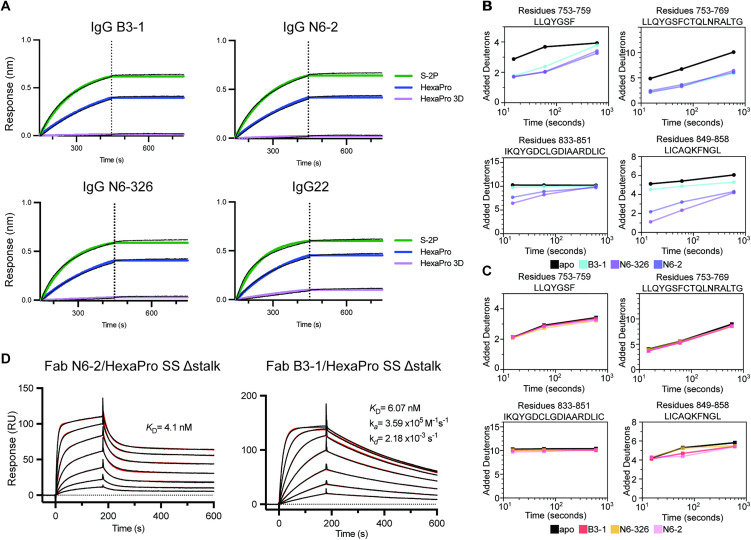
S2 apex-directed antibodies bind to open-interface spike or S2 subunit but not to closed-interface prefusion spike conformations. (**A**) BLI binding analyses of S2 apex-directed antibodies to SARS-CoV-2 Wuhan-1 variant spike ectodomain with varying propensities for trimer opening and breathing. Reference-subtracted binding curves are shown in black; fitted curves are overlaid in color. (**B**) HDX-MS analysis of S2 apex-directed antibody IgGs (N6-2, B3-1, and N6-326) reveals protection at two regions (residues 753–769 and residues 833–858) on the SARS-CoV-2 S-2P spike ectodomain in its open-interface conformation at 4 °C. (**C**) No protection was observed for the closed-interface prefusion conformation at 37 °C, indicating lack of binding to the fully closed prefusion spike. (**D**) SPR binding kinetics of N6-2 and B3-1 Fabs to the prefusion-stabilized S2-only subunit (HexaPro SS Δstalk). Experimental curves are shown in black with fitted data displayed in red. N6-2 was fit using a two-state binding model, whereas B3-1 was fit using a 1:1 binding model.

Hydrogen/deuterium exchange mass spectrometry (HDX-MS) studies have demonstrated that the S2 subunit of the SARS-CoV-2 spike protein becomes more solvent-accessible as protomers adopt open conformations within the trimeric structure [37]. This observation suggests that S2 apex-directed antibodies may preferentially recognize the open-interface conformation rather than the closed-interface conformation of the prefusion spike trimer. To assess the conformational sensitivity of binding, we performed BLI assays using IgGs B3-1, N6-2, and N6-326, along with IgG22, a previously reported stem-helix-directed antibody [23] (Fig 2A). In a single-concentration BLI experiment, all four antibodies exhibited the highest binding response to the two-proline-stabilized spike (S-2P), which more readily adopts an open conformation [37]. Binding signal was reduced for the six-proline-stabilized spike (HexaPro), which favors a closed prefusion conformation [35,37], and was abrogated entirely for the HexaPro 3-down variant (‘HexaPro 3 down’ or ‘HexaPro 3D’), which incorporates engineered disulfide bonds (S383C/D985C) to lock all RBDs in the down conformation, thus exclusively adopting a closed conformation [37,38]. These findings indicate that the epitopes recognized by these S2 apex-directed antibodies are likely occluded in the fully closed prefusion spike trimer and become accessible only in more open spike conformations or in isolated S2 subunits.

To assess the conformational specificity of the S2 apex-directed antibodies, we performed HDX-MS on the S-2P spike ectodomain under conditions that favor either the open-interface or the closed-interface prefusion conformations. Conformational bias was induced by pre-incubating the S-2P spike at either 4 °C to enrich for open-interface trimer (>90%) or at 37 °C to favor the closed-interface prefusion state (>90%) [37]. HDX-MS analyses were performed in the presence or absence of each antibody. Under the open-interface conditions (4 °C), all three antibodies—B3-1, N6-2, and N6-326—conferred localized protection from hydrogen-deuterium exchange relative to the no-antibody-added apo S-2P control (Fig 2B, 2C). No protection was observed under closed-interface conditions (37 °C), indicating that antibody binding is exclusive to the open-interface trimer conformation. HDX-MS mapping revealed that all three S2 apex-directed antibodies protected two discrete regions within the S2 subunit: residues 753–769 (amino acid sequence: LLQYGSFCTQLNRALTG) within the upper helix (UH, residues 743–779) of S2 and residues 849–858 (amino acid sequence: LICAQKFNGL) within the fusion peptide proximal region (FPPR, residues 828–853). Notably, B3-1 conferred less protection within the FPPR relative to N6-2 and N6-326, suggesting fewer contacts or weaker binding in this region. Together, these findings indicate that the antibodies target a shared epitope spanning the upper helix and FPPR of S2 in the open-interface trimer, though their specific interactions likely differ. The absence of exchange protection under closed-interface prefusion conditions (Fig 2C) confirms that epitope accessibility requires transient opening of the spike trimer.

### Structural characterization of the SARS-CoV-2 S2 prefusion trimer apex-directed antibodies

**Fig 3:**
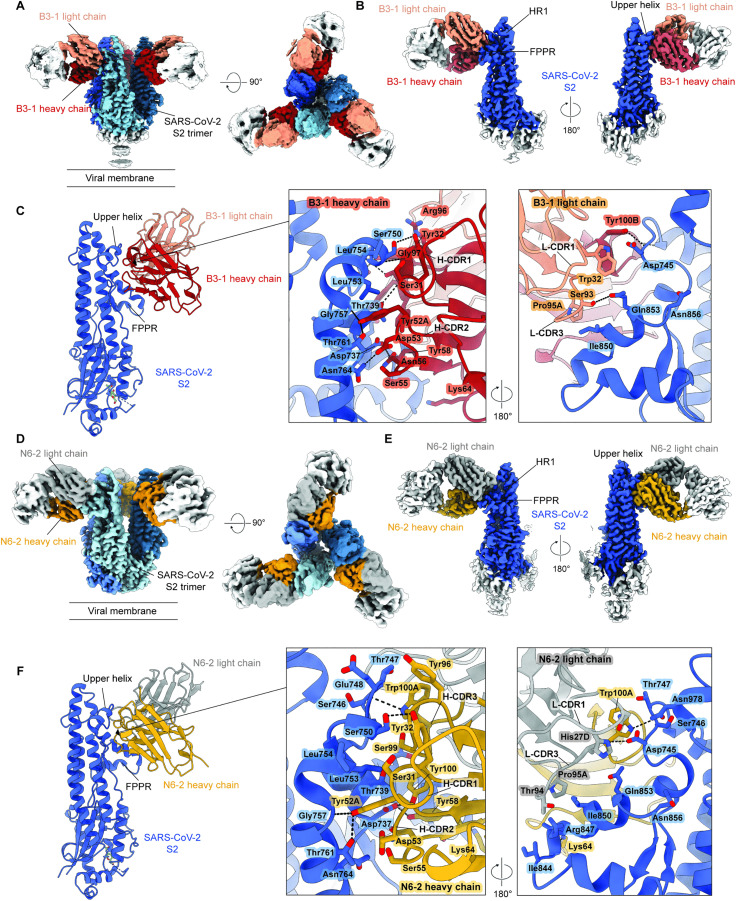
Cryo-EM structures of S2 apex-directed antibodies B3-1 and N6-2 reveal a cryptic epitope at the apex of the SARS-CoV-2 S2 trimer. (A, D) Top-down and side views of global cryo-EM reconstructions of antibodies B3-1 (**A**) and N6-2 (**D**) bound to the stabilized S2 trimer, HexaPro SS. **(B, E)** Cryo-EM local refinement maps highlighting detailed Fab binding interface to a single S2 protomer for B3-1 (**B**) and N6-2 **(E)**. **(C, F)** Close-up views showing heavy and light chain CDR loops of B3-1 (**C**) and N6-2 (**F**) engaging residues within the upper helix and fusion peptide proximal region of the S2 subunit.

To evaluate binding affinities, we measured the interactions of N6-2 and B3-1 antigen-binding fragments (Fabs) with a stabilized S2-only construct lacking the C-terminal helical stalk (HexaPro SS Δstalk, residues 697–1141) using surface plasmon resonance (SPR). Both Fabs bound to HexaPro SS Δstalk with high affinity, exhibiting dissociation constants (*K*_D_) of 4.1 nM for N6-2 and 6.1 nM for B3-1 (Fig 2D). Fab N6-326, which shares the same heavy chain sequence as N6-2, displayed weaker affinity, with a *K*_D_ of 29.4 nM (S3A Fig). Therefore, N6-2 was prioritized for downstream structural characterization. Although N6-2 exhibited single-digit nanomolar affinity for the HexaPro full spike (S3B Fig), cryo-EM analysis did not yield interpretable 2D classes or 3D reconstructions from the collected datasets. Therefore, we used a prefusion-stabilized SARS-CoV-2 S2 subunit construct for subsequent cryo-EM studies. To gain structural insights into epitope recognition, we performed cryo-EM on Fab–HexaPro SS Δstalk complexes. 6,012 micrographs were collected on a 300 kV Titan Krios for the B3-1 complex, yielding a final stack of 301,860 particles and a 3.4 Å-resolution 3D reconstruction. For the N6-2 complex, 2,763 micrographs were collected on a 200 kV Glacios, yielding a final stack of 599,302 particles and a 3.1 Å-resolution 3D reconstruction (Figs 3A, 3D; S4, S5; Table 1). In both maps, the stabilized S2 trimer adopted an open-interface prefusion conformation, which enhanced epitope accessibility and permitted Fab engagement at the membrane-distal apex. Notably, the two Fab–S2 complexes exhibited subtle differences in apex opening, consistent with the dynamic “breathing” behavior of the S2 subunit [24,37,39,40]. Symmetry expansion and local refinement of the Fab–S2 binding interfaces further improved map quality, yielding resolutions of 3.2 Å for the B3-1 complex and 2.9 Å for the N6-2 complex (S4, S5, S6 Fig**s**; Table 1).

**Table 1:** EM data collection and refinement statistics.

Structural model	SARS-CoV-2 S2 + Fab B3-1		SARS-CoV-2 S2 + Fab N6-2	
**EM data collection**				
EMDB	EMD-73731	EMD-73730	EMD-73735	EMD-73732
Microscope	Titan Krios		Glacios	
Voltage (kV)	300		200	
Detector	Gatan K3		Falcon 4	
Magnification (nominal)	29,000		29,000	
Pixel size (Å/pix)	0.81		0.94	
Exposure rate (e^-^/pix/sec)	10.6		2.55	
Exposure (e^-^/Å^2^)	80.5		40	
Defocus range (μm)	1.5-2.5		1.0-2.5	
Tilt angle (°)	30		0	
Micrographs collected	6,012		2,763	
Micrographs used	4,336		2,538	
Particles extracted (total)	2,020,014		1,584,826	
**3D reconstruction statistics**				
	Global	Focused	Global	Focused
Particles	301,860	905,580	599,302	1,797,906
Symmetry	C3	C1	C3	C1
Map sharpening B-factor	-158.2	-155.0	-111.2	-75.5
Unmasked resolution at 0.5 FSC (Å)	3.9	4.1	4.2	4.4
Masked resolution at 0.5 FSC (Å)	3.6	3.6	3.5	3.3
Unmasked resolution at 0.143 FSC (Å)	3.7	3.8	3.7	3.7
Masked resolution at 0.143 FSC (Å)	3.4	3.2	3.1	2.9
**Model refinement and validation statistics**				
PDB ID		9Z12		9Z18
Amino acids		568		577
RMSD bonds (Å)		0.005		0.004
RMSD angles (º)		0.81		1.03
Average amino acid B-factors		65.5		48.8
Ramachandran				
Favored (%)		95.5		98.4
Allowed (%)		4.5		1.6
Outliers (%)		0		0
Rotamer outliers (%)		0.83		0.41
Clash score		4.72		3.84
C-beta outliers (%)		0		0
CaBLAM outliers (%)		1.81		1.43
CC (mask)		0.77		0.77
MolProbity score		1.55		1.17
EMRinger score		3.11		4.23

Both B3-1 and N6-2 showed heavy chain-dominant interactions with the UH and FPPR of the S2 subunit (Fig 3B). For B3-1, the heavy chain buries 742 Å^2^ of surface area on each S2 protomer, with key interactions including hydrogen bonds mediated by H-CDR2 and H-CDR3 loops with UH residues. Additionally, the B3-1 framework region 3 (residues 58–64) engaged the FPPR, enhancing interface stability. Specifically, H-CDR2 residue Tyr52A (Kabat numbering) formed hydrogen bonds with UH residues Gly757 and Thr761, while Tyr58 interacted with UH residue Asp737. H-CDR3 residue Tyr100B mediates another hydrogen bond with the backbone of UH residue Asp745 (Fig 3C). The light chain contributes an additional 299 Å^2^ of buried surface area, primarily through L-CDR1 and L-CDR3 loops at the FPPR. A notable contact includes a hydrogen bond between L-CDR3 residues Ser93 and S2 residue Gln853. Importantly, several residues within or adjacent to the B3-1 epitope correspond to known mutation sites observed in SARS-CoV-2 VOCs. For instance, Asn764 (mutated to Lys in VOCs including BA.1, XBB.1.16, and FD.1.1) forms a hydrogen bond with B3-1 H-CDR2 residue Asp53. Additionally, the sidechain of Asn856 (mutated to Lys in Omicron BA.1) lies approximately 9 Å from the nearest B3-1 contact residue, Gln853 (S6C Fig). The proximity of these substitutions to the B3-1 epitope suggests potential immune selection pressure or a structural role in modulating spike protein stability.

N6-2 binds the apex of S2 in a manner similar to B3-1. The N6-2 heavy chain, primarily through H-CDR2 and H-CDR3, buries 704 Å^2^ of surface area on each S2 protomer, while the light chain buries an additional 300 Å^2^ (Fig 3D-3E). Like B3-1, the H-CDR3 loop of N6-2 (residues 97–100A) interacts with UH residues 746–750 located near the apex of the S2 trimer. Notably, S2 residue Asn764, which is mutated to Lys in several VOCs as described above, interacts with H-CDR2 residues Asp53 and Ser55 (Fig 3F). The L-CDR3 loop of N6-2 also engages FPPR residues 844–853, a region that is proximal to Asn856, another VOC mutation site (S6D Fig).

To further evaluate the impact of the Omicron-specific spike substitutions on antibody binding, we measured the affinities of N6-2 and B3-1 Fabs to S2-only constructs bearing Omicron BA.1 substitutions using SPR. The N6-2 Fab exhibited a 13-fold reduction in binding affinity to the Omicron BA.1 S2 protein compared to the Wuhan-1 variant (Figs 2D; S7A). Similarly, B3-1 Fab displayed a 10.4-fold reduction in affinity for the Omicron BA.1 S2 construct (Figs 2D; S7B). Qualitative BLI analysis of IgG binding to spike ectodomains from multiple SARS-CoV-2 variants shows that, at the same concentration, antibodies N6-2 and B3-1 have the greatest binding response to Wuhan-1 (HexaPro) and Alpha variant spikes. Binding was progressively reduced for Beta and Delta variants, with the greatest reduction observed for Omicron BA.1 (S7C, S7D Figs). To further quantify binding, we performed an SPR experiment by immobilizing the SARS-CoV-2 HexaPro Omicron BA.1 spike ectodomain and measuring binding to serially diluted concentrations of N6-2 Fab (600–18.75 nM). Consistent with the qualitative BLI analysis, the resulting signal was too low to determine a *K*_D_ for N6-2 Fab binding to the Omicron spike (S7E Fig). To pinpoint specific amino acid substitutions responsible for diminished binding, we employed mammalian surface display of spike proteins containing individual amino acid substitutions in the context of a panel of VH3–30 antibodies. Strikingly, all antibodies exhibited markedly reduced binding to spikes harboring the N856K substitution within the FPPR region (S7F Fig).

### S2 prefusion trimer apex-directed antibodies weakly neutralize rVSV-SARS-CoV-2 Wuhan-1 but not authentic SARS-CoV-2 VOCs

**Fig 4:**
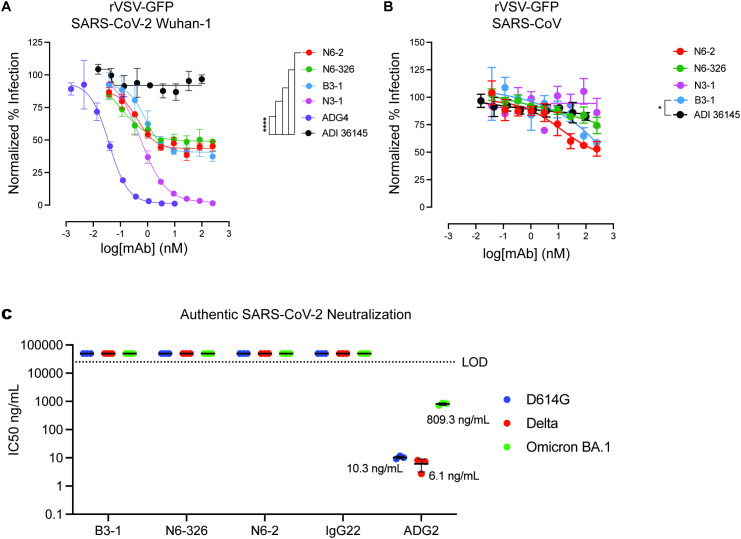
S2 apex-directed antibodies weakly neutralize SARS-CoV-2 pseudovirus but fail to neutralize the authentic virus. **(A, B)** Neutralization of rVSV-GFP pseudoviruses bearing SARS-CoV-2 Wuhan-1 (**A**) or SARS-CoV (**B**) spike proteins by S2 apex-directed antibodies. Area under the curve (AUC) was calculated, and statistical significance was assessed using one-way ANOVA with Tukey’s multiple comparisons test (**A**) or Kruskal-Wallis one-way ANOVA (**B**), depending on normality and homoscedasticity. **** p < 0.0001, * p < 0.05. (**C**) Neutralization of authentic SARS-CoV-2 variants (D614G, Delta, and Omicron BA.1) by S2 apex-directed antibodies (B3-1, N6-2, and N6-326) showed no detectable activity.

We evaluated the neutralization activity of S2 apex-directed antibodies using replication-competent vesicular stomatitis virus (rVSV) pseudotypes bearing SARS-CoV-2 (Wuhan-1) and SARS-CoV spike proteins [41]. The S2 apex-directed antibodies exhibited detectable but modest neutralizing activity against rVSV-SARS-CoV-2 (Wuhan-1) (Fig 4A), whereas an unrelated monoclonal antibody targeting Crimean–Congo hemorrhagic fever virus (ADI-36145 [42]) showed no effect. In comparison, the RBD-directed control antibodies ADG4 [14] and N3-1 [32] potently neutralized the same pseudovirus. Given the high sequence conservation at the S2 apex (S8 Fig), we also assessed the neutralization activity against rVSV-SARS-CoV (Fig 4B). N6-2 and N6-326 showed no appreciable neutralization activity, whereas B3-1 exhibited weak but statistically significant neutralization relative to the negative control.

We next evaluated the neutralizing activity of N6-326, B3-1, and N6-2 against SARS-CoV-2 variants D614G, Delta, and Omicron BA.1, using an authentic live-virus neutralization assay with Vero E6-C1008 cells, which we chose for their commercial availability and the ability to standardize findings across multiple laboratories (Fig 4C). In addition to these S2 apex-directed antibodies, we included the S2 stem helix-directed antibody IgG22 [23] as a negative control and the broadly neutralizing RBD-directed antibody ADG2 [13] as a positive control. As expected, ADG2 effectively neutralized the SARS-CoV-2 D614G and Delta variants but showed reduced potency against Omicron BA.1. The negative control IgG22 showed no neutralizing activity against any tested SARS-CoV-2 variant, consistent with previous reports [23]. In contrast to the weak neutralization observed in the rVSV neutralization assays, the S2 apex-directed antibodies (B3-1, N6-2, and N6-326) failed to neutralize the authentic SARS-CoV-2 D614G, Delta, or Omicron BA.1. These findings suggest that, in authentic virus contexts, the S2 epitope recognized by these antibodies has reduced accessibility, limiting their neutralizing activities.

### Prophylactic administration of S2 apex-directed antibody N6-2 in mice showed no protection against mouse-adapted SARS-CoV-2 MA10 challenge

**Fig 5:**
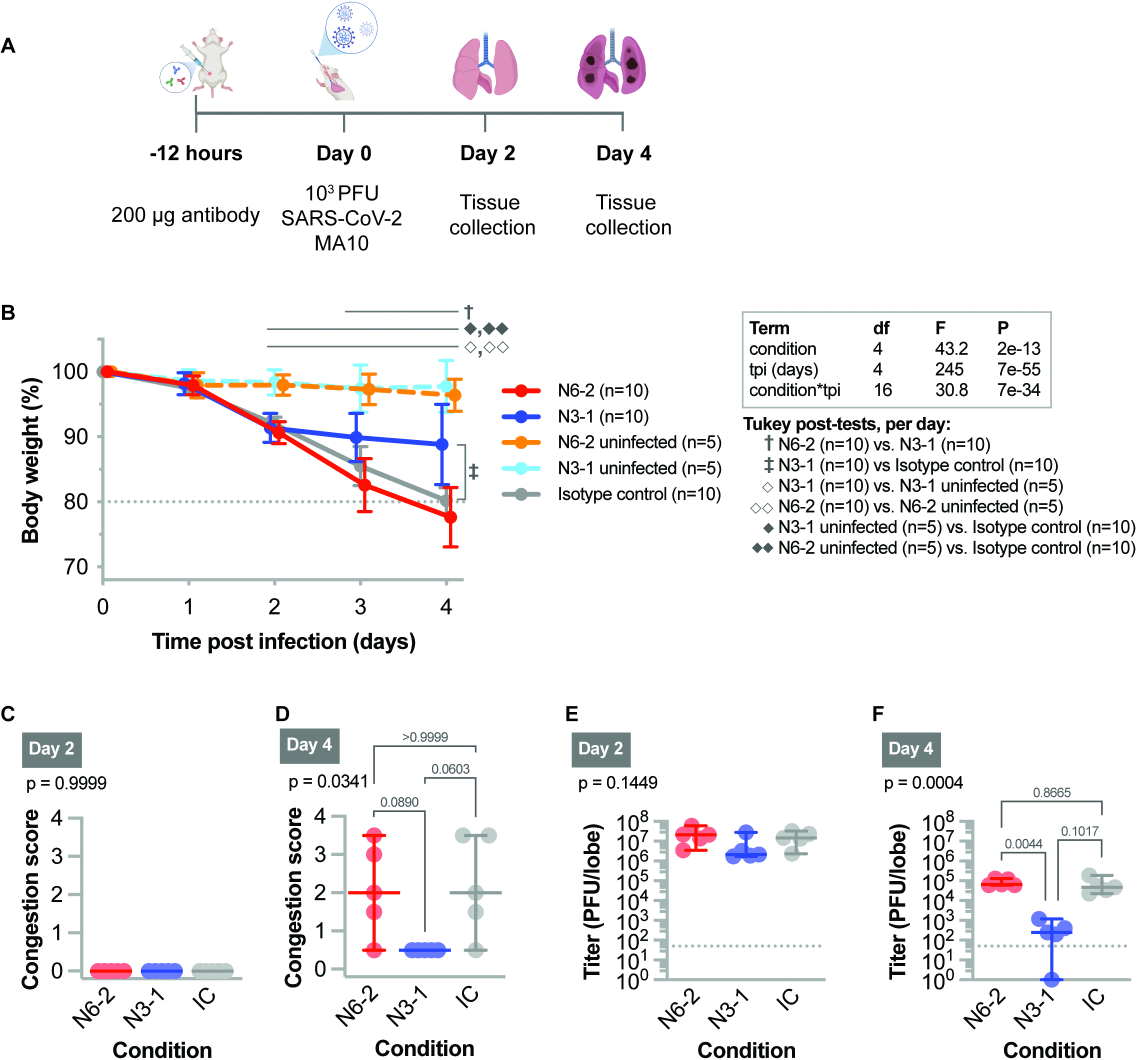
S2 apex-directed antibody N6-2 alone fails to protect mice from SARS-CoV-2 MA10 challenge. (**A**) Schematic of the experimental design for the mouse challenge model, detailing the timeline for prophylactic antibody administration, infection with mouse-adapted SARS-CoV-2 MA10, and subsequent tissue collection and evaluation. Created in BioRender. Leist, S. (2026) https://biorender.com/8faw01s (**B**) Mouse body weights were monitored daily following the indicated challenge. Percentage body weight loss over time post-infection (tpi) for each treatment is shown as a line plot, summarized by the mean ± 95% confidence interval (CI). Changes in body weight were analyzed using mixed-effects model regression with random effects for individual mice (repeated measures), with degrees of freedom (df), *F*-statistic, and corresponding *P*-value reported for condition, tpi, and their interaction. Tukey post hoc tests were used for pairwise comparisons per day; results with *P* < 0.05 are indicated by symbols (defined in the key), with horizontal lines denoting multiple consecutive days meeting the criterion. (**C-F**) Congestion scores (**C-D**) and lung viral titers (**E-F**) per condition at day 2 or day 4 are shown as dot plots, summarized by the median ± 95% CI. Due to the ordinal nature of congestion scores and non-normality of viral titers, data were analyzed using analysis of variance (ANOVA) on ranks per day, with the overall *P*-value listed for each panel. When ANOVA *P* < 0.05, pairwise comparisons were performed using Dunn’s post hoc test and reported accordingly.

To evaluate the protective efficacy of S2 apex-directed antibodies in vivo, we prophylactically treated 12-month-old female BALB/c mice with 200 µg of the S2 apex-directed antibody N6-2, RBD-directed antibody N3-1, or an isotype control. Antibodies were administered 12 hours prior to intranasal infection with 10^3^ PFU of mouse-adapted SARS-CoV-2 MA10 [43]. Dedicated groups of mice were sacrificed on day 2 and day 4 post-infection to evaluate macroscopic changes in lung discoloration (congestion scores) and determine viral load in the lungs over the course of infection (schematic Fig 5A). Mice treated with N6-2 or the isotype control experienced rapid weight loss, approaching 20% by day 4 post-infection, indicative of severe disease. In contrast, N3-1-treated mice exhibited a more modest weight loss, which stabilized after day 2 post-infection. By day 4, mice receiving N3-1 alone exhibited a peak weight loss of 11.2% (88.8% of baseline weight), suggesting partial protection. These results suggest that prophylactic administration of the non-neutralizing S2 apex-directed antibody N6-2 does not confer protection in this mouse model. No macroscopic changes were evident on day 2 post-infection across all groups (Fig 5C). By day 4, N6-2- and isotype-treated mice exhibited high variability in lung congestion scores, ranging from 0.5 to 3.5. In contrast, N3-1-treated animals showed minimal lung discoloration (score: 0.5) (Fig 5D). Viral load quantification in the inferior lung lobe revealed no significant differences between groups on day 2 post-infection (Fig 5E). By day 4, viral loads in N6-2- and isotype-control-treated animals averaged 10^5^ PFU/tissue. In contrast, N3-1-treated animals exhibited a ~ 200-fold reduction (average viral load: 5.1x10^2^ PFU), with 1 of 5 animals showing no detectable viral load (Fig 5F).

Control groups were included for each treatment condition, in which mice received prophylactic antibody administration but were mock-infected with PBS rather than virus, allowing assessment of treatment effects on all phenotypes in the absence of the infection. All mock-infected mice were sacrificed on day 4 after mock infection. As expected, no changes in body weight, lung congestion scores, or live viral titer in the lungs were observed in these groups, confirming that the observed phenotypes were virus dependent (S1 Data). Collectively, these results indicate that the S2 apex-directed antibody N6-2 alone does not confer protection against body weight loss or lung pathology in the SARS-CoV-2 MA10 mouse challenge model.

## Discussion

In this study, we used yeast surface display to isolate a panel of S2 apex-directed antibodies from convalescent individuals infected with the SARS-CoV-2 Wuhan strain. Initial attempts to structurally characterize these antibodies using cryo-EM with stabilized spike ectodomains were unsuccessful, likely due to epitope occlusion by the S1 subunit. To overcome this limitation, we used a prefusion-stabilized S2-only construct [30], which enabled high-resolution structural characterization of the cryptic epitope targeted by these antibodies. Cryo-EM reconstructions revealed that two high-affinity Fabs (KD < 10 nM) bind the apex of the S2 subunit near the fusion peptide proximal region (residues 834–857), engaging the epitope primarily through heavy-chain interactions. Biophysical analyses using BLI and HDX-MS demonstrated that these antibodies selectively recognize the open-interface conformation of the spike protein. Binding was abolished when the spike protein was conformationally stabilized in the closed state, either through engineered interprotomeric disulfide bonds or by incubation at 37 °C. Despite the high sequence conservation of this epitope among sarbecoviruses, neutralization assays using rVSVs and authentic virus showed weak or undetectable neutralizing activity. Consistent with the poor neutralizing activity, prophylactic administration of these antibodies in a murine challenge model failed to confer protection against SARS-CoV-2 infection.

The lack of neutralizing activity and protective efficacy of the antibodies characterized in this study is somewhat surprising given they target the apex of the S2 fusion machinery. This stands in contrast to findings for other class I viral fusion proteins, such as the fusion (F) proteins from human respiratory syncytial virus (RSV) [44–47] and human metapneumovirus (hMPV) [48–51], where the apex region of the fusion machinery is a well-established site of vulnerability. In these viruses, the apex is highly neutralization-sensitive due to the substantial conformational rearrangements required for membrane fusion. Although the FPPR of SARS-CoV-2 S2 undergoes analogous structural transitions (S10 Fig), we speculate that the lack of neutralizing activity observed here arises from limited epitope accessibility on the native virion surface. This interpretation is supported by our HDX-MS findings, which demonstrate that the cryptic epitope targeted by these antibodies is only exposed when the spike protein is incubated at 4 ºC—a condition that promotes opening of the spike protomers. In contrast to the SARS-CoV-2 spike, the F proteins of RSV, hMPV, and other paramyxoviruses lack an S1-equivalent fusion-suppressive cap, rendering their fusion machinery apex more accessible to antibodies on the virion surface. These structural differences likely explain the divergent neutralization profiles observed for apex-targeting antibodies across viral families.

Previous studies of SARS-CoV-2 spike-directed antibodies have mainly focused on epitopes within the RBD and NTD [11–15,52], with comparatively fewer reports describing antibodies that target the S2 subunit. Existing S2-directed antibodies include those recognizing the fusion peptide [19–22], the helical stalk [23–25,53–55], the S2 apex [26–29], a fully closed prefusion S2 trimer [29], and, most recently, reported during the preparation of this manuscript, an antibody that binds postfusion S2 [26] (S9 Fig). Despite growing interest, the mechanisms underlying neutralization and protection by S2-targeted antibodies remain incompletely understood, and large regions of the S2 subunit surface area remain antigenically uncharacterized. Notably, antibodies targeting the helical stalk have demonstrated reactivity and potent neutralization across sarbecoviruses, whereas those directed at the fusion peptide exhibit broad neutralization due to the high sequence conservation of this critical region for membrane fusion. The mechanisms that govern the differences in neutralization and protective capacity between fusion peptide-directed antibodies and S2 apex-directed antibodies remain unclear. ACE2 binding has been shown to expose the fusion peptide and enhance binding of antibodies that target this region of S2. It is possible that the S2 apex remains occluded at physiological temperature even with ACE2 bound, preventing neutralization. Interestingly, organoid-based assays that evaluate neutralization potency in relevant tissue have shown neutralization activity for S2-directed antibodies previously characterized as protective but non-neutralizing [56]. These assays offer a valuable approach for exploring mechanisms of antibody-mediated protection in different contexts. Some weakly or non-neutralizing S2 antibodies have been shown to provide *in vivo* protection through Fc-mediated effector functions [21]. However, *in vitro* ADCP and ADCT activation by the S2 apex-directed, non-neutralizing antibody 54043–5 did not translate into protection from SARS-CoV-2 infection in mice [29]. Additional experiments are necessary to determine the precise mechanisms that prevent neutralization and the protective capacity of the antibodies described in this study, as well as the extent to which their neutralization activity depends on the cell lines used and their TMPRSS2 expression levels. The primary focus of this work is the structural characterization of a cryptic S2 epitope and the associated spike conformational dynamics, providing a framework for understanding antibody accessibility, informing functional antibody engineering, and guiding the design of next-generation immunogens.

Several studies have reported enrichment of S2-directed antibody lineages following SARS-CoV-2 infection [27,57–59]. Our results suggest that the cryptic epitope characterized here at the prefusion trimer apex is transiently exposed during infection, enabling elicitation of such antibodies. However, the precise mechanism of exposure remains unclear—whether it occurs through conformational “breathing” of the spike trimer or partial unfolding of the protein. Previous studies have shown that stabilizing the S2 subunit in its prefusion trimer conformation results in immunogenicity comparable to that of the full spike protein [30,39,60]. These findings indicate that the S2 subunit represents a promising immunogen due to the preservation of neutralizing epitopes reported at the fusion peptide [19–22] and helical stalk [23–25] regions; however, increased accessibility to the S2 prefusion apex does not appear to enhance immunogenicity [30,60]. This lack of improved immunogenicity is in contrast to other class I fusion proteins such as RSV F [44,61], where stabilizing F in its prefusion trimer conformation preserved neutralizing epitopes at the prefusion apex. Our study characterizes cryptic epitopes at the prefusion S2 apex and provides a potential explanation for this observation, as the S2 prefusion apex–directed antibodies characterized here are non-neutralizing and non-protective.

A deeper understanding of spike protein conformational dynamics and the temporal exposure of transient epitopes could inform the rational design of next-generation vaccines. For instance, glycan shielding is a well-established strategy to mask unwanted epitopes and redirect the immune response toward more protective sites [62,63]. S2-based immunogens could incorporate glycosylation motifs at the apex to reduce elicitation of non-protective antibodies. In parallel, structure-based vaccine design approaches, such as molecular dynamics simulations, could be employed to further stabilize the spike trimer in a closed conformation, thereby limiting exposure of the cryptic apex epitope [39]. The S2 apex-directed antibodies reported in this study provide valuable tools to probe spike conformational dynamics and epitope accessibility, offering mechanistic insights into viral entry and guiding the design of more effective immunogens.

## Methods

### Ethics statement

The collection of blood specimens from convalescent individuals was conducted under approval from the University of Texas at Austin Institutional Review Board (protocol 2020-03-085; *Breadth of serum antibody immune responses prior to, or following, patient recovery in asymptomatic and non-severe COVID-19*). Informed written consent was obtained from all participants. The generation and tissue culture use of all rVSV-CoVs were conducted at biosafety level 2 (BSL-2) in compliance with protocols approved by the Environmental Health and Safety Department and Institutional Biosafety Committee at Albert Einstein College of Medicine.

All animal work was approved by the Institutional Animal Care and Use Committee at the University of North Carolina at Chapel Hill under protocol 20–144, following guidelines outlined by the Association for the Assessment and Accreditation of Laboratory Animal Care (AAALAC) and the U.S. Department of Agriculture.

### Yeast display strains and media

Yeast strain EBY100 (MATa AGA1::GAL1-AGA1::URA3 ura3–52 trp1 leu2-delta200 his3-delta200 pep4::HIS3 prbd1.6R can1 GAL) was acquired from ATCC (Cat. No. MYA-4941) and used for antibody surface display and selection. To enhance antibody surface expression, the human chaperone BiP (binding immunoglobulin protein) and yeast PDI (protein disulfide isomerase) were genomically integrated as an expression cassette at the HO locus. Yeast cultures were grown in rich medium (YPD; Takara, Cat. No. 630409) or in selective medium for leucine prototrophy following library transformation (Takara, Cat. No. 630310). YEP-galactose was used for expression of displayed antibody libraries (1% yeast extract, 1% bacto-peptone, 0.5% NaCl, 2% galactose, 0.2% glucose).

### Yeast display antigens and antibodies

Stabilized spike soluble ectodomain antigen with proline substitutions at positions 986 and 987 (S-2P) was expressed and purified using methods as previously described [34]. The purified protein was biotinylated using the EZ-link kit (Thermo Scientific, Cat. No. 21435) and subsequently labeled with streptavidin-AF647 (Invitrogen, Cat. No. S32357). Stabilized S2 antigens were labeled with anti-StrepTagII-AF647. Fab library light chains were labeled with anti-FLAG M2-FITC (Sigma-Aldrich, Cat. No. F4049).

### Donors

Blood samples were collected from three PCR-confirmed patients at either day 12 (donors 1 and 2) or day 11 (donor 3) post-symptom onset. None of the donors required hospitalization or experienced severe disease. Peripheral blood mononuclear cells (PBMCs) and plasma were isolated via density gradient centrifugation using Histopaque-1077 (Sigma-Aldrich, Cat. No. 10771–100ML).

### Library assembly and bacterial transformation

Donor B-cell VH and VL amplicons were amplified via PCR to include adapters for cloning into yeast expression vectors. Assembly into the yeast kappa and lambda expression vectors was performed using Golden Gate assembly in 20 μL reactions. Each reaction contained 2 μL of 10x AarI buffer (Thermo Fisher Scientific, Cat. No. B27), 0.4 μL of 50x oligo buffer (Thermo Fisher Scientific, Cat. No. ER1582), 0.2 μL of 100 mM ATP (Thermo Fisher Scientific, Cat. No. R0441), 20 fmol backbone DNA, 40 fmol VH and VL amplicons, 0.5 μL (2 U/μL) of AarI endonuclease (Thermo Fisher Scientific, Cat. No. ER1582), and 0.5 μL of T7 ligase (3000 U/μL) (NEB, Cat. No. M0318). Assemblies were scaled up to 16 reactions in 8-well strips and thermocycled at 37 °C for 15 minutes, followed by 74 cycles of 37 °C for 2 minutes and 16 °C for 1 minute. The reaction concluded with 37 °C for 60 minutes, 80 °C for 15 minutes, and a hold at 4 °C. Following thermocycling, reactions were consolidated and column purified using Promega Binding Solution (Promega, Cat. No. A9303) to bind DNA to Zymo-spin II columns (Zymo Research, Cat. No. C1008). The columns were washed twice with DNA Wash Buffer (Zymo Research, cat. no. D4003) and eluted in 30 μL nuclease-free water. For library transformations, DH10B *E. coli* cells were diluted 1:100 from a confluent culture into 50 mL Superior broth (AthenaES, Cat. No. 0105). When cells reached an OD_600_ of 0.4-0.6, they were washed 3 times with cold 10% glycerol and resuspended to a final volume of 600 μL. The purified library was added to cells and electroporated at 2.5 kV using an *E. coli* Pulser electroporator (Bio-Rad) using Genepulser 0.2 cm cuvettes (Bio-Rad, Cat. No. 1652086), with 200 μL per transformation.

### Library transformation into yeast and protein expression

Purified libraries were linearized for integration into the yeast genome via homologous recombination at the Leu2 locus. For each 1 μg library plasmid, 0.5 μL NotI (10 units/μL) (NEB, Cat. No. R0189) was used in 10 μL reactions with the supplied Buffer 3.1. Reactions were incubated overnight at 37 °C and heat-inactivated at 80 °C for 20 minutes. The digests were pooled, column-purified as previously described, and eluted in 25 μL of nuclease-free water. Electroporation was performed using 10 μg or 20 μg of linearized DNA. We determined that 10 μg of linearized DNA was sufficient for generating library sizes of ~10^6^, while library sizes exceeding 10^7^ could be achieved with 20 μg DNA. Transformed yeast were recovered in SD-Leu medium (see “Strains and Media” section) and passaged once at 1:100 to reduce contamination from untransformed cells. For Fab library expression, yeast were washed in YEP-galactose (see “Strains and Media”) and diluted 1:10 to a final volume of 10 mL. Induction was carried out for 48 hours at 20 °C with shaking.

### Fab library labeling and selection

Expressed yeast libraries were harvested in 100 μL volumes, representing approximately 10^7^ cells, and washed with PBSA buffer (1x PBS, 2 mM EDTA, 0.1% Tween-20, 1% BSA, pH 7.4). Antigen was incubated with the cells in 1 mL in PBSA at a concentration of 200 nM for 1 hour at room temperature (RT), followed by washing with PBSA at 4 °C. Cells were then labeled with secondary antibodies (mouse anti-human FITC, 1:100; streptavidin-AF647, 1:100; mouse anti-human Fc-AF647, 1:50). After labeling, cells were washed twice and resuspended in 2 mL of cold PBSA for sorting. Cell sorting was performed using a Sony SH800 fluorescent cell sorter. For first-round libraries, sorted cells were collected into 2 mL SD-Leu medium supplemented with penicillin/streptomycin (Gibco, Cat. No. 15140122) and recovered by shaking incubation for 1–2 days. Recovered cells were either used for subsequent rounds of selection or plated directly for phenotypic characterization of clones.

### Phenotyping assays

Sorted clones from the second or third round of selection were picked into microplates as previously described [64]. Following antibody expression, 10 μL of cells were transferred to a fresh 2-mL microplate and washed twice with 200 μL cold PBSA buffer. Cells were labeled with 200 nM spike or S2 antigen in 50–100 μL PBSA at room temperature for 1 hour with shaking at 1000 rpm (3 mm orbit). After antigen labeling, cells were washed twice, and secondary labeling was performed as previously described [64]. Labeled cells were resuspended in 200 μL of ice-cold PBSA immediately prior to analysis. Samples were analyzed using a Sony SA3800 Spectral Cell Analyzer.

### Long-read sequencing

Sequencing libraries were prepared from amplicon samples using the Native Barcoding Kit (Oxford Nanopore Technologies, Cat. No. EXP-NBD104) in combination with the Ligation Sequencing Kit (Oxford Nanopore Technologies, Cat. No. SQK-LSK109), following the manufacturer’s instructions. Four sequencing libraries were pooled per flow cell and sequenced on four GridION flow cells (Oxford Nanopore Technologies; R9.4.1) for 72 hours using a GridION Mk1 device (Oxford Nanopore Technologies; Oxford, UK). Raw sequencing data were base-called live using the high-accuracy model in Guppy (v.4.0.11).

### Sequence processing and consolidation into VH-VL clones

We implemented a bioinformatic pipeline to accurately derive VH-VL sequences from MinION and iSeq data and estimate the relative abundance of individual VH-VL pairs within each YSD round. Antibody V(D)J annotation of raw MinION reads was performed using MiXCR (v3.0.13) [65]. Sequence clusters were iteratively adjusted by growing or shrinking based on annotated features from each read, followed by sequence error correction and consolidation within clusters. High-quality Illumina reads were optionally incorporated to improve accuracy. VH-VL clones were then defined for each sample, and their relative abundances were quantified by counting the number of reads mapped to each clone. Only reads with a length of 1700–2100 base pairs were included in the final quantification to ensure consistency.

### Monoclonal antibody expression and purification

VH-VL candidates were cloned into custom Golden Gate-compatible pCDNA3.4 vectors for IgG1 expression. For transfections, VL plasmid DNA was mixed with the corresponding VH plasmid DNA at a 3:1 ratio and transiently transfected into Expi293F cells (Invitrogen, Cat. No. A14635) following the manufacturer’s recommended protocol. Monoclonal antibodies were harvested 5–7 days post-transfection. Expi293F cells were centrifuged at 300 × g for 5 min, and the supernatants were collected and further centrifuged at 3,000 × g for 20 min at 4 °C. The resulting supernatants were diluted to a 1x PBS final concentration and passed through Protein G or Protein A agarose affinity column (Thermo Scientific, Cat. Nos. 20003 or 20422). The flow-through was collected and reapplied to the column three times. Columns were washed with 10 column volumes (CV) of PBS, and antibodies were eluted using 5 mL of 100 mM glycine (pH 2.5) into a neutralization buffer containing 500 μL 1 M Tris-HCl (pH 8.0). Antibodies were buffer exchanged in PBS (pH 7.4). For Fab preparation, IgGs N3-1, N6-326, B3-1, and N6-2 were incubated overnight with Lys-C (Thermo Scientific, Cat. No. 90051) and purified using the CaptureSelect IgG-CH1 Affinity Matrix column (Thermo Scientific, Cat. No. 1943200250). The Fab fragments were further purified by size-exclusion chromatography on a Superdex 200 Increase 10/300 GL column (Cytiva; 28-9909-44) eluted in a buffer containing 2 mM Tris (pH 7.5), 200 mM NaCl, and 0.03% NaN_3_. Peak fractions were pooled and concentrated using Amicon Ultra Centrifugal Filters, 30 kDa MWCO (Millipore, Cat. No. UFC9030).

### ELISA

Antigen ELISA plates were prepared using high-binding microplates (Corning, Cat. No. 3366) coated with antigen diluted in PBS to a final concentration of 2 μg/mL. A total of 50 μL of antigen solution was added to each well and incubated overnight at 4 °C with shaking at 100 rpm (3 mm orbit). Plates were blocked with PBSM (2% milk in PBS) for 1 hour at room temperature. Following blocking, plates were washed three times with 300 μL PBS-T (0.1% Tween-20). Purified antibodies were prepared at a starting concentration of 10 μg/mL in PBSM and serially diluted. Antibodies were added to the plates and incubated for 1 hour at RT. Plates were then washed three times with PBS-T, and a secondary goat anti-human Fab-HRP antibody (Sigma-Aldrich, Cat. No. A0293) was applied at 1:5,000 dilution in PBSM (50 μL per well) and incubated for 45 min at RT. HRP substrate (50 μL) was added to wells, and the reaction was allowed to proceed for 5–15 minutes before being quenched with 50 μL 4 M H_2_SO_4_. Absorbance at 450 nm was measured using a plate reader.

### Spike protein purification

Previously published mammalian expression plasmids encoding stabilized SARS-CoV-2 S2 subunit—HexaPro SS (residues 697–1141 with additional stabilizing substitutions as previously described [30])—were transiently transfected into FreeStyle 293F cells (Thermo Fisher, Cat. No. R79007) using 25 kDa linear polyethylenimine (PEI). These constructs included a C-terminal foldon trimerization motif from T4 fibritin, an HRV3C cleavage site, an 8x His-tag, and a Strep-tag II [30]. Sterile-filtered kifunensine was added 3 h post-transfection to a final concentration of 5 μM. Supernatants were harvested and filtered on day 6, followed by affinity column purification using Strep-Tactin Sepharose resin (IBA, Cat. No. 2-1201-002). Elution was performed with 1x Strep-Tactin elution Buffer E (IBA, Cat. No. 2-1000-025), and the samples were concentrated for size-exclusion chromatography using a Superose 6 Increase 10/300 GL column (Cytiva, Cat. No. 17-5172-01). Samples were eluted into a buffer containing 2 mM Tris (pH 7.5), 200 mM NaCl, and 0.03% NaN_3_. Major peak fractions were pooled and further concentrated using Amicon Ultra Centrifugal Filter, 30 kDa MWCO (Millipore, Cat. No. UFC9030).

Plasmids (pαH) encoding stabilized MERS-CoV S-2P (GenBank ID: YP_007188579.1), SARS-CoV S-2P (GenBank ID: UUW06557.1), and WIV-1 S-2P (GenBank ID: AGZ48828.1) were transiently transfected into FreeStyle 293F cells (Thermo Fisher, Cat. No. R79007) using the same method as described above. Supernatants were harvested on days 6–7 post-transfection, filtered, and purified on Strep-Tactin Sepharose resin (IBA, Cat. No. 2-1201-002). Elution was performed using the same Buffer E, followed by size-exclusion chromatography on a Superose 6 Increase 10/300 GL column (Cytiva, Cat. No. 17-5172-01). Peak fractions were eluted into 2 mM Tris (pH 7.5), 200 mM NaCl, and 0.03% NaN_3_, and samples were concentrated using the same spin column as described above.

### Hydrogen-deuterium exchange

Prior to hydrogen exchange, purified SARS-CoV-2 S-2P was diluted to 3.33 μM trimer and incubated at 4 °C for 4 days to favor the open-interface trimer conformation or at 37 °C for 16 hours to favor the closed-interface prefusion conformation [37]. Before initiating exchange, S-2P was further diluted to 1.67 μM trimer (5 μM monomer), and antibody was added at a 1.25:1 molar ratio of antibody IgG to monomer (6.25 μM antibody) to ensure saturation. Samples were incubated for 10 minutes to allow antibody binding prior to hydrogen exchange. For all hydrogen exchange experiments, deuterated buffer was prepared by lyophilizing PBS (pH 7.4; Sigma-Aldrich, Cat. No. P4417) and reconstituting it in D_2_O (Sigma-Aldrich, Cat. No. 151882). Continuous exchange experiments were initiated by diluting samples 10-fold into temperature-equilibrated (25 °C) deuterated PBS buffer (final spike trimer concentration of 0.167 μM; pH_read_ 7, pD 7.4). At the indicated time points, samples were quenched by mixing 60 μL of the partially exchanged protein with 60 μL of 2x quench buffer (3.6 M GdmCl, 500 mM TCEP, 200 mM glycine, pH 2.4) on ice. Samples were incubated on ice for 1 minute to facilitate partial unfolding and proteolytic degradation before being flash-frozen in liquid nitrogen and stored at -80 °C. Quenching time points were set at 15 seconds, 60 seconds, and 600 seconds.

### Protease digestion and LC/MS

LC-MS was conducted as previously described [37]. Briefly, samples were thawed immediately prior to injection into a cooled valve system (Trajan LEAP) coupled to an LC (Thermo UltiMate 3000). Sample time points were injected in random order. The valve chamber, trap column, and analytical column were maintained at 2 °C, while the protease column was held at 10 °C. Quenched samples underwent inline digestion using two immobilized acid proteases in series: aspergillopepsin (Sigma-Aldrich, Cat. No. P2143) and porcine pepsin (Sigma-Aldrich, Cat. No. P6887), at a flow rate of 200 μL/min of buffer A (0.1% formic acid). Protease columns were prepared in-house by coupling protease to beads (Thermo Scientific POROS 20 AL aldehyde activated resin, Cat. No. 1602906) and manually packing them into columns (2 mm ID × 2 cm, IDEX C-130B). Post-digestion, peptides were desalted for 4 minutes on a manually packed trap column (Thermo Scientific POROS R2 reversed-phase resin, Cat. No. 1112906, 1 mm ID × 2 cm, IDEX C-128). Peptide separation was performed using a C8 analytical column (Thermo Scientific BioBasic-8 5 μm particle size 0.5 mm ID × 50 mm 72205–050565) with a gradient elution of buffer B (100% acetonitrile, 0.1% formic acid). The gradient consisted of a 5–40% increase in Buffer B over 14 minutes at a flow rate of 40 μL/min, followed by a 40–90% increase over 30 seconds. Trap and analytical columns were subjected to a sawtooth wash and equilibrated at 5% buffer B before subsequent injections. Protease columns were washed with two injections of 100 µL of 1.6 M GdmCl, 0.1% formic acid between injections. Peptides were eluted directly into a Q Exactive Orbitrap Mass Spectrometer operating in positive mode (resolution 70,000; AGC target 3e6; maximum IT: 50 ms; scan range 300–1500 m/z). A tandem mass spectrometry experiment was performed on one undeuterated sample to identify peptides. Full MS settings were the same as described above, with the following dd-MS^2^ settings: resolution 17,500; AGC target 2e5; maximum IT: 100 ms; loop count: 10; isolation window: 2.0 m/z; normalized collision energy (NCE): 28; charge states 1 and ≥7 excluded; dynamic exclusion: 15 seconds. LC and MS methods were executed using Xcalibur 4.1 software (Thermo Scientific).

### Peptide identification

Byonic (Protein Metrics) was used to identify unmodified and glycosylated peptides in the tandem mass spectrometry data. The search library included the full sequence of the expressed construct, encompassing the signal sequence, tags and trimerization domain. Sample digestion parameters were set to non-specific. Precursor and fragment mass tolerances were set to 6 ppm and 10 ppm, respectively. Variable N-linked glycosylation was enabled, with a library of 132 human N-glycans included in the search. No non-glycosylated peptides corresponding to any of the 22 known glycosylation sites in the spike sequence were observed, regardless of the glycosylation search parameters. Peptide lists containing sequence, charge state, and retention time were exported from Byonic and imported into HDExaminer 3 (Sierra Analytics). When multiple peptide lists were generated, all were imported and combined within HDExaminer 3 for further analysis.

### HDExaminer 3 analysis

Peptide isotope distributions at each exchange time point were fit in HDExaminer 3. For glycosylated peptides, only the highest-confidence modification was included in the mass spectral search and subsequent analysis. Deuteration levels were calculated by subtracting the mass centroids of undeuterated peptides from those of deuterated peptides.

### Cryo-EM sample preparation and data collection

Fabs N6-2 and B3-1 were co-eluted with HexaPro SS via SEC at a concentration of ~0.5 mg/mL in crystal buffer (2 mM Tris, pH 7.5, 200 mM NaCl, and 0.03% NaN_3_). A 3 μL aliquot of the complexed samples was applied to plasma-cleaned Protochips Au-Flat GF-1.2/1.3 grids (Part No. GF-1.2/1.3-3AU-45nm-50) and plunge-frozen using a Vitrobot Mark VI (Thermo Scientific) with a 4-second blot time, blot force of -2, 100% humidity, and a temperature of 22 °C.

The Fab N6-2–HexaPro SS complex was imaged on a 200 kV Glacios microscope (Thermo Scientific) at a pixel size of 0.94 Å/px and an electron exposure of 40 e^-^/Å^2^ in electron counting mode. The Fab B3-1–HexaPro SS complex was imaged on a 300 kV Krios microscope (Thermo Scientific) at a pixel size of 0.81 Å/px and an electron exposure of 80 e^-^/Å^2^ in electron counting mode.

### Cryo-EM data processing and model building

Micrographs were processed using CryoSPARC [66] v2, v4.4.1, and v4.5.1 through a series of steps, including motion correction, CTF-estimation, particle picking, 2D classification, ab-initio reconstruction, heterogeneous refinement, homogeneous refinement, and non-uniform refinement. To enhance the local resolution of Fab binding interfaces, each Fab/HexaPro SS structure underwent local refinements with masks generated in UCSF ChimeraX [67,68] (detailed processing workflows are described in S4 and S5 Figs). Initial Fab models were constructed using SAbPred [69]: ABodyBuilder with a Kabat numbering scheme. These models were iteratively refined using ISOLDE [70], Coot [71], and Phenix [72].

### Surface plasmon resonance for antibody binding affinity measurements

His-tagged HexaPro SS construct was immobilized to approximately 200 response units (RU) on Ni-NTA sensor chips (GE Healthcare, Cat. No. BR100407) using a running buffer of 10 mM HEPES, 150 mM NaCl, and 0.05% (v/v) Tween-20 at pH 7.4. Fabs, at concentrations ranging from 1.56 to up to 400 nM, were injected into the flow cell. The binding data for B3-1 and N6-326 interacting with the SARS-CoV-2 HexaPro SS constructs were fit using a 1:1 binding model, whereas the N6-2–HexaPro SS interaction was best described by a two-state binding model. In contrast, binding data for both N6-2 and B3-1 interacting with the Omicron SS construct were fit using a 1:1 binding model.

### Biolayer interferometry for spike variant binding comparisons

Antibody binding to various spike variants was assessed using Anti-hIgG Fc Capture (AHC) Biosensors (Sartorius, Cat. No. 185063) on the Octet RED96e (FortéBio) system. Antibodies were captured to a level of 0.6 nm and subsequently dipped into wells containing 100 nM spike protein. Binding curves were reference-subtracted using buffer controls, and association and dissociation kinetics were fitted using Octet Data Analysis software v11.1.

### Cell culture

Vero cells (ATCC, CCL-81) were cultured in Dulbecco’s Modified Eagle’s Medium (DMEM, high glucose; Gibco, Cat. No. 11965126) supplemented with 2% heat-inactivated fetal bovine serum (FBS; Bio-Techne), 1% penicillin-streptomycin (Thermo Fisher Scientific, Cat. No. 15140148), and 1% GlutaMAX (Thermo Fisher Scientific, Cat. No. 35050–061).

### Automated pipeline for spike variant cloning

A high-throughput automated cloning pipeline was employed to assemble spike and S2 variants using Golden Gate cloning. The system incorporated an Echo 525 acoustic liquid handler (Beckman Coulter), a Tecan Fluent robotic liquid handler (Tecan), and a QPix 420 Colony Picker (Molecular Devices). Golden Gate assembly–compatible parts, including IDT eBlocks or plasmids (Addgene, Cat. No. 172727–172733), were arranged in a 384-well Echo Source Plate (PP-0200) and transferred to 96-well PCR destination plates using the Echo 525.

Golden Gate reactions (10 µL per well) were prepared by combining 0.25 µL T7 DNA Ligase (NEB M0318S), 0.25 µL AarI (Thermo Fisher, Cat. No. ER1582), 0.20 µL AarI Oligo (Thermo Fisher), 1 µL T4 DNA Ligase Buffer (NEB B0202S), 1 µL of each part (eBlock or plasmid), 1 µL sfGFP-DO destination vector (Addgene, Cat. No. 172721–172726), and nuclease-free water. The reactions were subjected to 25 cycles of digestion (37 °C for 1 min) and ligation (16 °C for 2 min), followed by a 30-min digestion step at 37 °C and a final inactivation step at 80 °C for 20 min.

### Transformation and colony picking

For high-throughput transformations, 50 µL of Zymo DH10β Mix & Go Competent Cells (Zymo T3019) were dispensed into 96-well PCR plates. Aliquots (4 µL) of the Golden Gate reactions were transferred to corresponding wells, mixed gently, and incubated at 4 °C for 10 min. The resulting DNA–cell mixtures (54 µL) were transferred into deep-well grow blocks (Axygen P-2ML-SQ-C-S) containing 150 µL Superior Broth (AthenaES, Cat. No. 0105) per well and incubated at 37 °C for 1 h on a plate shaker at 950 rpm. Following incubation, 5 µL of each outgrown culture was spotted onto Nunc OmniTrays (Thermo Fisher, Cat. No. 140156) containing LB agar supplemented with 100 µg/mL carbenicillin. After drying at room temperature, the trays were incubated at 37 °C for 12–16 h. White (nonfluorescent) colonies were screened and picked using the QPix 420 (Molecular Devices) to ensure the presence of spike inserts rather than sfGFP alone. Selected colonies were transferred into deep-well blocks containing 1 mL Superior Broth with 100 µg/mL carbenicillin and cultured overnight at 37 °C with shaking. Bacterial cultures were pelleted by centrifugation at 3,000 × g for 10 min, and plasmid DNA was purified using a Tecan Fluent robotic liquid handler and the Promega Wizard SV 96 Plasmid DNA Purification Kit (Promega, Cat. No. A2250). Clones were verified by Sanger sequencing.

### HEK293T transfection

HEK293T cells (ATCC, Cat. No. CRL-3216) were seeded into 6-well plates (0.3 × 10^6^ cells/mL) or 12-well plates (0.1 × 10^6^ cells/mL) one day prior to transfection. Upon reaching 60–80% confluence, cells were transfected with spike expression plasmids using Lipofectamine 3000 (Invitrogen, Cat. No. L3000015) and Opti-MEM (Gibco, Cat. No. 51985091) according to the manufacturer’s protocol. A 3:1 ratio of Lipofectamine 3000 to plasmid DNA was used. Cells were harvested or assayed 48 h post-transfection.

### Mammalian display flow cytometry and data analysis

HEK293T cells were harvested 48 h post-transfection and washed once with PBS before gentle resuspension in PBS containing 1% BSA and 2 mM EDTA at pH 7.4 (PBS-BSA). Cell density was measured using a LUNA-II Automated Cell Counter (Logos Biosystems, Cat. No. L40002), and cells were centrifuged at 200 × g for 1 min. After decanting the supernatant, cells were resuspended at 3 × 10^6^ cells/mL in chilled PBS-BSA.

Flow cytometry assays were prepared in deep-well blocks (Axygen, P-2ML-SQ-C-S) by adding 50 µL of the cell suspension (1.5 × 10^5^ cells) to wells containing 1 µg/mL Mouse anti-FLAG M2 antibody (Sigma-Aldrich, Cat. No. F3165) and predetermined concentrations of primary antibody in PBS-BSA. The mixtures were incubated at room temperature for 1 h with shaking (950 rpm), centrifuged at 500 × g for 2 min in a swinging-bucket rotor, and washed twice with PBS-BSA. Secondary antibody solutions (500 µL) containing 5 µM Alexa Fluor 488 anti-mouse (SouthernBiotech, Cat. No. 1031-30) and 10 µM Alexa Fluor 647 anti-human (SouthernBiotech, Cat. No. 2048-31) were added, and samples were incubated in the dark at 4 °C for 25 min with shaking (950 rpm). After washing twice, cells were resuspended in 300 µL PBS-BSA for analysis on a SA3800 Spectral Cell Analyzer (SONY).

Unstained HEK293T cells were used to define scatter and fluorescence gates. Singlet discrimination was performed using forward scatter-height (FSC-H) vs. forward scatter-area (FSC-A) and side scatter-height (SSC-H) vs. side scatter-area (SSC-A). At least 10,000 singlet events per sample were acquired. Alexa Fluor 488 (AF-488) and Alexa Fluor 647 (AF-647) signals were recorded, and spectral unmixing was applied to correct for spillover and autofluorescence. Data analysis was conducted in FlowJo v9 (BD).

Spike expression levels were quantified using the median height (H) AF-488 signal (anti-FLAG), and normalized measurements for spike variants (x) expression values relative to wild-type (6P-D614G) were calculated using the formula:

${Normalizedexpression=Log}_{\text{2}}(Median:\text{4}\text{8}\text{8}\_H_{x}/Median:\text{4}\text{8}\text{8}\_H_{\text{6}P\_D\text{6}\text{1}\text{4}G})$

For binding measurements, the AF-488 signal was used as an internal normalization control. Normalized binding values were calculated as:

$Normalizedbinding={Log}_{\text{2}}\left(\frac{Median:\text{6}\text{4}\text{7}\_H_{x}/Median:\text{4}\text{8}\text{8}\_H_{x}}{Median:\text{6}\text{4}\text{7}\_H_{\text{6}P\_D\text{6}\text{1}\text{4}G}/Median:\text{4}\text{8}\text{8}\_H_{\text{6}P\_D\text{6}\text{1}\text{4}G}}\right)$

### Generation of rVSV-CoV-2 Wuhan-1 and rVSV-CoV-1

A plasmid encoding the VSV antigenome was modified to replace its native glycoprotein G with the CoV spike of interest. CoV spike sequences were obtained from GenBank (SARS-CoV-2 Wuhan, GenBank MN908947.3, and SARS-CoV, GenBank NC004718.3). Each CoV spike was engineered with a 19 amino acid C-terminal deletion, as previously described for rVSV-SARS-CoV-2 [41]. An enhanced green fluorescent protein (eGFP) reporter gene was incorporated into the VSV antigenome as a separate transcriptional unit. Plasmid-based rescue of the rVSVs was performed as previously described [41,73]. Briefly, 293FT cells were transfected with the VSV antigenome plasmid and helper plasmids encoding T7 polymerase and VSV N, P, M, G, and L proteins, using polyethylenimine as the transfection reagent. Supernatants from transfected cells were transferred to Vero cells 48 hours post-transfection. The appearance of eGFP-positive cells indicated successful virus production. Viruses were plaque-purified on Vero cells and propagated via cell subculture. RNA was isolated from viral supernatants of plaque-purified virus, and the spike gene sequence was confirmed by Sanger sequencing. Viral stocks were concentrated by ultracentrifugation at 28,000 rpm for 4 hours in an SW28 rotor, aliquoted, and stored at -80 °C.

### rVSV-CoV microneutralization assay

Antibody was prepared at the following initial concentrations: ADI-36145 (100 nM), ADG4 (10 nM), N6-2 (250 nM), N6-326 (250 nM), B3-1 (250 nM), and N3-1 (250 nM). Serial 3-fold dilutions were performed in DMEM supplemented with 2% FBS. Antibodies were incubated with rVSV-SARS-CoV-2 (Wuhan-1) or rVSV-CoV for 1 hour at room temperature. Media was removed from Vero cells seeded in 96-well plates (Corning, Cat. No. 3595), and 65 µl of the virus/antibody mixture was added to each well. Cells were incubated at 37 °C with 5% CO_2_ for 10 hours. Subsequently, cells were fixed with 4% paraformaldehyde, washed with 1x PBS, and stored in 1x PBS containing Hoechst 33342 (Invitrogen, Cat. No. H3570) at a dilution of 1:4,000. Viral infection was quantified by automatic enumeration of GFP-positive cells from captured images using a Cytation 5 automated fluorescence microscope (BioTek), with analysis performed using Gen5 data analysis software (BioTek). Each experimental repeat was processed independently by subtracting the corresponding background noise and normalizing the Optical Densities (OD) relative to a positive control. Background noise was inferred by considering the average blank OD readout within the corresponding plate. Denoised and normalized OD values were jointly processed to fit a single sigmoidal curve per individual time point using the least-squares minimization function as implemented in the SciPy package [74]:


$$\begin{array}{c} y=y_{min}+\left(y_{max}-y_{min}\right)/\left[\text{1}+{\text{1}\text{0}}^{\left(\left({log}_{\text{1}\text{0}}{EC}_{\text{5}\text{0}}-x\right)\times Hill\right)}\right] \end{array}$$

Where *y* corresponds to the OD (denoised and normalized); *y*_min_ and *y*_max_ are the minimum and maximum ODs, respectively; EC_50_ denotes the antibody titer that achieves half-maximum absorbance, *y*_max_; Hill describes the slope of the curve; and *x* is the log_10_ (1/dilution). Neutralization was quantitatively assessed by calculating the area under the curve (AUC) of the fitted sigmoidal curves. Each antibody group was tested for normality using the Anderson-Darling test and homoscedasticity using Bartlett’s test. Parametric and non-parametric tests were run accordingly. All statistical analyses were conducted using GraphPad Prism software.

### Live virus neutralization assays

Reverse genetics were employed to recover full-length SARS-CoV-2 (D614G [75], Sequence Accession No. MT020880), SARS-CoV-2 Delta (Accession No. OV116969.1), and SARS-CoV-2 Omicron (Accession No. EPI_ISL_6647961) viruses expressing nanoluciferase (nLuc). Neutralization assays were adapted from previously reported protocols [76] to optimize the system for evaluating a diverse panel of SARS-CoV-2 variants. Vero E6 cells (ATCC CRL-1586) were plated at 2 × 10^4^ cells per well in 96-well black-bottomed cell plates (Corning, Cat. No. 3916) 24 hours prior to experiment. Antibodies were standardized to 1 mg/mL, diluted 1:20 in a non-binding dilution plate with virus growth medium (1x MEM; Gibco, Cat. No. 11095080, supplemented with 5% FBS; Hyclone, Cat. No. SH30070.03HI, and 1% Pen-Strep; Gibco, Cat. No. 10378016), and serially diluted 3-fold across 8 dilutions. At BSL-3, nLuc-expressing viruses were individually diluted in virus growth medium and combined 1:1 with the antibody dilutions in the dilution plates. The plates were incubated for 1 hour at 37 °C with 5% CO_2_. The virus-antibody mixtures were then transferred to black-bottomed cell plates for a final virus dilution of 800 PFU/well and a starting antibody concentration of 25,000 ng/mL. Plates were incubated at 37 °C with 5% CO_2_ for 24 hours. Luciferase activity was measured using the Nano-Glo Luciferase Assay System (Promega, Cat. No. N1130). Cells were lysed, and enzymatic activity was quantified using a GloMax Plate Luminometer (Promega, Cat. No. GM3500). Inhibitory Concentrations at 50% neutralization (IC_50_) were defined as the dilution corresponding to a 50% reduction in relative light units (RLU) compared to “Virus and Cell” and “Virus-only” control wells. ID_50_ values were calculated using an Excel macro and analyzed using GraphPad Prism (10.1.1). The monoclonal antibody ADG2 [14] was included as a performance control to standardize assay conditions.

### *In vivo* antibody treatment and challenge

Twelve-month-old female BALB/cAnNHsd (BALB/c) mice were purchased from Envigo (Stock #047) and housed in the animal facility at the University of North Carolina at Chapel Hill until the start of the experiment. All *in vivo* challenge experiments were conducted in animal biosafety level 3 (BSL-3) facilities at the University of North Carolina at Chapel Hill. Mice were moved into the BSL-3 laboratory seven days prior to the virus challenge. Prophylactic treatment was administered 12 hours before infection via intraperitoneal injection of 200 µg of antibody (N6-2, N3-1, or isotype control), diluted in PBS to a total volume of 100 µL. All mice (n = 5/ treatment/ harvest day) were infected with 10^3^ plaque-forming units (PFU) of mouse-adapted SARS-CoV-2 MA10 [43] under anesthesia using a mixture of ketamine and xylazine. Mice were monitored daily for changes in body weight and clinical signs of disease. Statistical analyses, except for mixed models, were performed in GraphPad Prism version 10.0.1 (GraphPad Software, Boston, Massachusetts, USA, www.graphpad.com). Mixed models were analyzed using JMP Pro version 17.2.0 (JMP Statistical Discovery, Cary, North Carolina, USA, www.jmp.com), with repeated measures per subject controlled as a random variable. Fig legends provide details on biological replicates, statistical tests, and post-hoc analyses. On days 2 and 4 post-infection, five mice per treatment group were sacrificed for tissue collection. Macroscopic changes in lung discoloration were recorded for each animal as congestion scores. Inferior lung lobes were collected into vials containing PBS and glass beads and stored at -80 °C until use in plaque assays to determine viral loads. Congestion scores and lung titer data were analyzed using ANOVA with Dunn’s pairwise comparisons, as detailed in the figure legends. Additionally, five animals from each treatment group were mock-infected with PBS and sacrificed on day 4 post-infection to assess the effects of antibody treatment in the absence of virus infection.

### Plaque assay

Viral loads were quantified using a plaque assay performed on the inferior lung lobes of each animal. Briefly, lung tissues were thawed in vials containing PBS and glass beads, followed by homogenization. Serial 1:10 dilutions of lung homogenate supernatants were generated by diluting 50 µl of homogenate in 450 µl PBS, with further serial dilutions prepared up to 1:1,000,000. Diluted samples were added to a monolayer of Vero E6 cells (ATCC CRL-1586) plated at 500,000 cells per well 24 hours prior to the assay. The plates were incubated for 1 hour at 37 °C with 5% CO_2_ to allow viral adsorption. Wells were then overlaid with 0.8% agarose in 2x cell media and incubated for three days at 37 °C with 5% CO_2_. Plaques were visualized by adding 2 mL of neutral red dye to each well and incubating for 3 hours.

## Supporting information

S1 Fig Germline V_H_ gene usage of IgGs selected via yeast surface display. Germline V_H_ gene usage patterns of antibodies isolated from three COVID-19 convalescent donors (Donors 1-3) after three rounds of selection against stabilized SARS-CoV-2 spike ectodomain. For each donor, an initial Fab library was generated and subjected to yeast surface display selection. Notably, IGHV3-30 was prominently over-represented among the enriched antibody population targeting the spike antigen.
(TIF)

S2 Fig ELISA analysis of VH3–30 antibodies binding to recombinant S2. Purified VH3–30 antibodies, identified through YSD selections against SARS-CoV-2 spike ectodomain protein, were evaluated for binding to the recombinant S2 subunit (HexaPro SS) using ELISA.
(TIF)

S3 Fig Surface plasmon resonance studies for N6-326 and N6-2 Fab. (A) A stabilized prefusion SARS-CoV-2 S2 trimer was immobilized on a Ni-NTA sensor chip to measure binding affinity of Fab N6-326. Data were fit using a 1:1 binding model. Experimental data are shown in black, and fitted curves are shown in red. (B) SARS-CoV-2 HexaPro full spike was immobilized on a Ni-NTA sensor chip, and Fab N6-2 was titrated to assess binding affinity in the context of the full spike ectodomain. Data were fit using a two-state binding model, which was necessary to account for conformational changes in the spike protein that are required for epitope accessibility.
(TIF)

S4 Fig Cryo-EM processing workflow for the Fab B3-1/HexaPro SS complex. Single-particle cryo-electron microscopy data were processed using CryoSPARC versions 4.4.1 and 4.5.1 [66]. The workflow included motion correction, contrast transfer function (CTF) estimation, automated particle picking, and 2D classification. *ab initio* reconstruction, multiple rounds of heterogeneous and homogeneous refinement, reference-based motion correction, and rebalance orientation jobs to obtain the global map. A mask was generated using UCSF ChimeraX [68] for subsequent local refinement to enhance the Fab-S2 interface. The final map was sharpened using DeepEMhancer [77].
(TIF)

S5 Fig Cryo-EM processing workflow for the Fab N6-2/HexaPro SS complex. Single-particle cryo-electron microscopy data were processed using CryoSPARC versions 2 and 4.4.1 [66]. The processing pipeline included motion correction, contrast transfer function (CTF) estimation, automated particle picking, and 2D classification. *Ab initio* reconstruction and multiple rounds of heterogeneous and homogeneous refinement were performed to obtain the global map. A mask was generated using UCSF ChimeraX [68] for subsequent local refinement to enhance the Fab-S2 interface. The final map was sharpened using DeepEMhancer [77].
(TIF)

S6 Fig Cryo-EM map-to-model agreement. (A, B) Transparent cryo-EM local refinement maps for B3-1 (A) and N6-2 (B) in complex with S2, with corresponding atomic models docked into the map. Right panels show zoomed-in views of the heavy and light chains engaging the S2 single protomer at the UH and FPPR (843–853) region. Nitrogen atoms are colored blue, oxygen atoms are red, and sulfur atoms are yellow. (C, D) Zoomed-in views of Omicron mutations N764K and N856K at the B3-1 and N6-2 heavy chain binding interfaces.
(TIF)

S7 Fig Reduced binding of the S2-directed antibodies to Omicron substitutions. (A, B) SPR binding analysis for Fabs N6-2 (A) and B3-1 (B) to the SARS-CoV-2 HexaPro SS construct bearing Omicron BA.1 substitutions (“Omicron SS”). Experimental binding curves are shown in black, with 1:1 fitted curves displayed in red. (C, D) BLI analysis of S2-directed IgGs to 6-proline-stabilized spike ectodomains from SARS-CoV-2 VOCs. Binding curves are shown in black, with fitted curves overlaid in color. (E) N6-2 Fab binding to the 6-proline-stabilized Omicron spike ectodomain. The binding responses obtained for the tested Fab concentrations (18.75–600 nM) were insufficient to determine the binding affinity. (F) Flow cytometry binding analysis of S2-directed antibodies to six-proline-stabilized full-length SARS-CoV-2 spike D614G proteins harboring individual Omicron S2 substitutions displayed on HEK293T cell surfaces. Normalized binding was calculated as previously reported [78].
(TIF)

S8 Fig B3-1 and N6-2 epitope sequence conservation among sarbecoviruses. Sequence conservation of the B3-1 and N6-2 epitopes across SARS-CoV-2 VOCs, SARS-like betacoronaviruses, as well as MERS-CoV, OC43, and HKU1 (left). Red dots indicate key contact residues. ConSurf [79] conservation analysis of the SARS-CoV-2 spike protein structure (right).
(TIF)

S9 Fig Schematic summary of known S2 epitopes. Sequence presentation of the SARS-CoV-2 S2 subunit highlighting the locations of previously characterized antibody epitopes. Representative antibodies targeting each region are annotated, including those recognizing the S2 apex (54043–5 [29]), fusion peptide (COV44–62, COV44–79, COV91–27 [20]), stem helix (CC40.8 [25], IgG22 [23], S2P6 [24]), and postfusion S2 (1871 [80]). Residue numbering corresponds to the SARS-CoV-2 spike protein sequence.
(TIF)

S10 Fig Superposition of the N6-2/HexaPro SS structure and the postfusion S2 structure. Cryo-EM structure of antibody N6-2 (orange and gray) bound to the prefusion-stabilized S2 protein (blue) is superimposed with one protomer of the cryo-EM structure of the postfusion spike protein (PDB ID: 8FDW [81]; Postfusion FPPR is highlighted in dark pink.)
(TIF)

S1 Data Raw data file containing the values for all plotted data. Each tab in the spreadsheet contains the raw data for a plot in the manuscript figures.
(XLSX)
