# Supplementary figures and images for: Structural and functional characterization of a conserved cryptic epitope on SARS-CoV-2 spike S2 subunit
Source: PLoS Pathog. 2026 Aug 3;22(8):e1014391. doi: 10.1371/journal.ppat.1014391 (PMC13432147; doi:10.1371/journal.ppat.1014391)

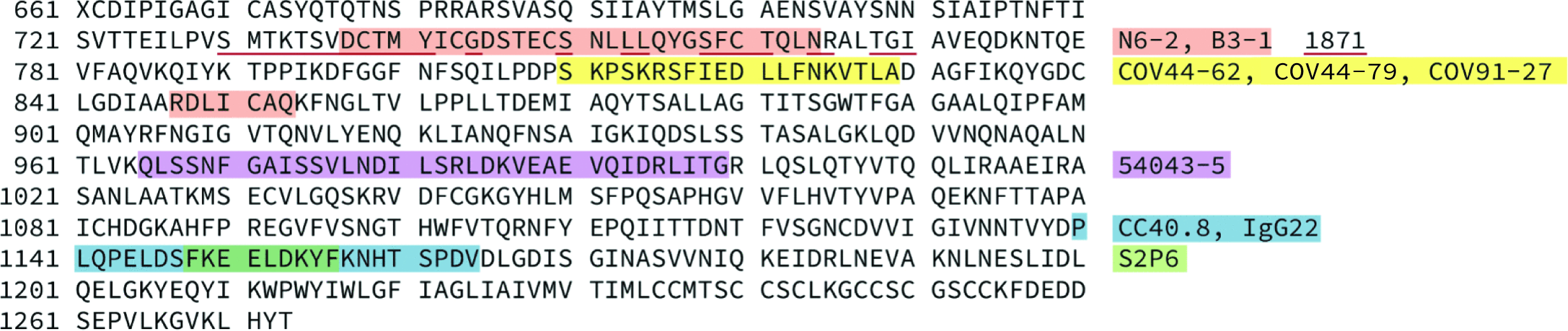

Supplement: S9 Fig — Sequence presentation of the SARS-CoV-2 S2 subunit highlighting the locations of previously characterized antibody epitopes. Representative antibodies targeting each region are annotated, including those recognizing the S2 apex (54043–5 [29]), fusion peptide (COV44–62, COV44–79, COV91–27 [20]), stem helix (CC40.8 [25], IgG22 [23], S2P6 [24]), and postfusion S2 (1871 [80]). Residue numbering corresponds to the SARS-CoV-2 spike protein sequence. (TIF) [file ppat.1014391.s009.tif]
